# Defect-Engineered MOF-808-SO_4_ as Efficient Solid Acid Catalysts for Esterification of n-Butyl Acetate

**DOI:** 10.3390/molecules31111908

**Published:** 2026-06-02

**Authors:** Wei Cao, Lifang Chen, Tingting Wang, Ke Wang, Zhen Song, Zhiwen Qi

**Affiliations:** State Key Laboratory of Chemical Engineering and Low-Carbon Technology, School of Chemical Engineering, East China University of Science and Technology, 130 Meilong Road, Shanghai 200237, China

**Keywords:** MOF-808, defect engineering, solid acid catalyst, esterification, n-butyl acetate

## Abstract

In order to address corrosion and pollution problems of liquid acids and limitations of traditional solid acids, sulfated MOF-808-SO_4_ catalysts were developed by creating unsaturated sites in MOF-808 for sulfate grafting with ligand defect engineering. Characterization verified framework integrity, successful sulfate coordination, and maintenance of high surface areas and tunable porosity. Temperature-programmed desorption of ammonia (NH_3_-TPD) establishes a clear consistent trend between defect density and the concentration as well as the strength of acid sites, indicating that a higher degree of ligand deficiency promotes the formation of more abundant and stronger acid centers. For esterification of acetic acid with n-butanol, the catalyst prepared by replacing 40 mol% of BTC with BDC achieved ≥99% conversion of acetic acid under mild conditions of 2.0 wt% catalyst loading and 1:2 alcohol/acid molar ratio at 120 °C for 6 h, outperforming conventional solid acids. This performance stems from high-density strong Brønsted acid sites strongly coordinated at defects and an open pore structure facilitating diffusion. The catalyst was easily recovered by ethanol washing and maintained stable activity over five cycles without loss of catalytic capability. This work suggests defect engineering as an effective strategy for tuning acidity and catalytic performance in MOF-based solid acids for green esterification.

## 1. Introduction

The esterification of acid and alcohol is one of the most important reactions in organic synthesis, fine chemical production, and biofuel preparation. The ester compounds produced via this reaction are widely used in industries as solvents, coatings, fragrances, plasticizers, pharmaceutical intermediates, and agrochemicals due to their favorable solubility, moderate volatility, and stable chemical properties [1]. Among them, n-butyl acetate, exhibits excellent dissolving ability and low-toxicity, environmentally friendly [2] characteristics, performing an irreplaceable role in industrial applications such as paint thinning, flavor blending, resin synthesis, and extraction separation [3]. With the growing demand for green chemicals and high-end fine chemicals, the development of efficient, stable, and environmentally friendly synthesis processes for n-butyl acetate has become an important research frontier in catalysis and materials chemical engineering.

In traditional industrial esterification processes, concentrated sulfuric acid is commonly used as a homogeneous catalyst. Although it exhibits high catalytic activity, it suffers from significant drawbacks, including severe corrosion of reaction equipment, difficulty in product–catalyst separation, complex post-treatment processes, generation of large amounts of acidic wastewater, and poor environmental compatibility [4]. To address these issues, researchers have turned to ion-exchange resins as heterogeneous solid acid catalysts [5,6]. Ion-exchange resins are recyclable and less corrosive, but they still face limitations such as low specific surface area, insufficient acid strength, and uncontrollable pore structures. In recent years, alternative catalytic systems such as ionic liquids have attracted considerable attention due to their unique properties, including negligible vapor pressure, high thermal stability, and tunable acidity [7,8,9]. However, their practical application is hindered by high cost, potential toxicity, and difficulties in product separation and catalyst recovery [10]. Consequently, researchers have further explored various heterogeneous solid acid catalytic systems, including molecular sieves, heteropoly acids, supported ionic liquids, and biomass-derived carbon-based acid catalysts [11]. These catalysts offer advantages such as facile separation, recyclability, and low corrosiveness. However, they still commonly face bottlenecks such as low specific surface area, uneven distribution of active sites, uncontrollable pore structures, and difficulty in precisely tuning acid strength, which limit their further applications in high-efficiency and high-selectivity esterification reactions.

Metal–organic frameworks (MOFs), a class of crystalline porous materials formed by the coordination self-assembly of metal clusters and organic ligands, exhibit great potential in heterogeneous catalysis due to their ultra-high specific surface areas, tunable pore structures, abundant active sites, and strong designability [12,13,14]. Through rational selection of metal centers and organic ligands, regulation of topological structures, functional modification, precise design of catalytic sites, pore environments, and diffusion pathways can be achieved at the molecular level, thereby significantly enhancing catalytic activity and selectivity [15,16]. Among numerous MOFs, zirconium-based MOFs have become promising platforms for acid-catalyzed reactions, attributed to their excellent hydrothermal stability, structural robustness, and abundant exposed active sites that are readily accessible to reactants [17,18,19], making them particularly suitable for reactions requiring medium to strong acid sites, such as esterification, hydrolysis [20], and isomerization [21,22].

MOF-808 is constructed from 6-connected Zr_6_ SBUs and 3-connected BTC linkers. With open metal sites, excellent structural stability, and good modifiability, it can be a promising platform for acid catalysis [23,24]. MOF-808-SO_4_ is obtained through post-synthetic sulfation treatment, whereby strong Brønsted acid sites (-HSO_4_) are introduced onto the zirconium clusters, significantly enhancing its acid catalytic performance. Thus, it exhibits excellent activity in reactions such as esterification, alkylation, and isomerization [18,25]. Additionally, the MOF-808 structure contains numerous adjustable ligand defect sites.

The introduction of defect ligands featuring distinct chain lengths or functional groups (such as monocarboxylic or dicarboxylic acids) enables the rational tuning of pore size distribution, pore volume, defect density, and spatial localization of acid catalytic sites [23,26,27]. Ma et al. [28] recently reported that by partially substituting the tricarboxylic acid ligand to construct defective MOF-808, additional acid-base sites were successfully introduced, increasing the conversion rate of furfural transfer hydrogenation from 72.9% to 94.7%. This strategy provides an important design concept to construct efficient solid acid catalysts with both size selectivity and diffusion advantages.

Despite significant progress in the acid catalysis of MOF-808 through defect engineering, current research still faces key challenges. On one hand, precise regulation of defect sites remains difficult. Salinger and Walton’s research [29,30] pointed out when defect sites were introduced via soft template method, due to the difficulty of MOF crystals effectively encapsulating large-sized template molecules, there is often a lack of clear structure–activity relationships between defect generation and pore regulation. On the other hand, the synergistic anchoring mechanism between defect sites and sulfate groups remains unclear [31]. Chu et al. [32] indicated that, although removing coordinated formic acid through acid treatment can form unsaturated coordinated metal sites during the synthesis of Ce-MOF-808, precise control of defect density and distribution remains challenging, and the generation of defect sites is often accompanied by changes in crystal stability. Currently, research on MOF-808-SO_4_ mostly focuses on individual regulation of pore structure and acidity through defects or sulfation, lacking the systematic understanding of synergistic mechanisms. Particularly for the probe reaction of n-butyl acetate synthesis, the intrinsic relationships between defect sites, sulfate anchoring, acid center construction, and catalytic performance still require further elucidation.

Herein, we utilize MOF-808 as the prototypical platform to carry out organic ligand defect engineering, realizing precise defect regulation of the density and three-dimensional distribution of coordinatively unsaturated sites. Subsequently, sulfate groups are grafted to fabricate a range of sulfated solid acid catalysts MOF-808-SO_4_. The influence of the defect ratio on the crystal structure, pore characteristics, acid-site distribution, and catalytic esterification performance of the materials is systematically studied. The key role of defect sites as anchoring sites for sulfate groups and platforms for constructing acid centers is clarified, aiming to provide a theoretical basis and new strategies for the design of green esterification catalytic systems.

## 2. Results and Discussion

### 2.1. Structures of the Defect Engineered MOF-808

**Figure 1:**
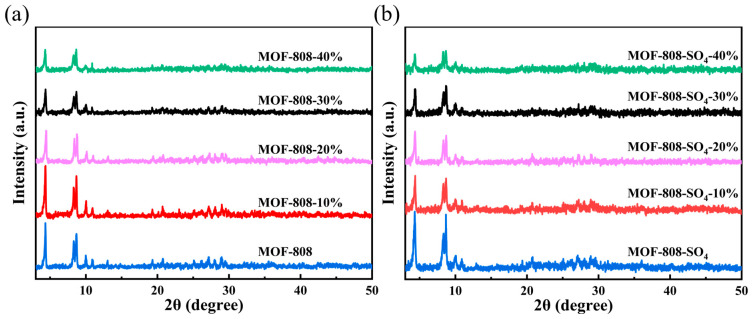
PXRD patterns with all MOF-808 (**a**) and MOF-808-SO_4_ samples (**b**).

A number of analytical technologies have been performed to gain information on MOF-808 including its structures and stability. As shown in Figure 1, all samples exhibit similar PXRD patterns, confirming the successful preparation of MOF-808 and MOF-808-SO_4_. The introduction of defective ligands and the post-synthetic modification process do not disrupt the structure of the parent MOF-808. Notably, before and after sulfation, as the proportion of defective ligands increases, the diffraction intensities of the characteristic peaks at 4.4° and 8.5° decrease, indicating that the crystallinity of the catalysts correspondingly decreases.

**Figure 2:**
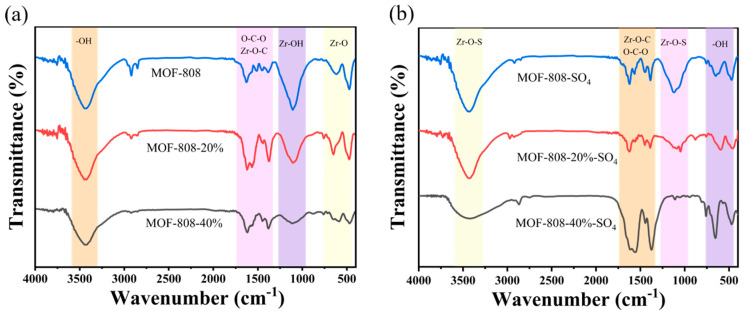
FT-IR spectra of MOF-808-A% (**a**) and MOF-808-A-SO_4_ (**b**), A = 0, 20, 40.

FT-IR spectroscopy was employed to identify the functional groups and investigate the coordination environment of the organic ligands within the framework. As illustrated in Figure 2a, MOF-808-X (X = 0%, 20%, 40%) exhibits characteristic peaks in the range of 1380–1620 cm^−1^. These peaks can be attributed to the symmetric stretching (-COO) at 1450 and 1371 cm^−1^, and the asymmetric stretching (-COO) at 1619 and 1550 cm^−1^ [33]. The vibration frequencies of the ligands are observed through bands at 1109 and 943 cm^−1^ [34]. The bands at 655 and 484 cm^−1^ correspond to Zr-O stretching vibrations [35]. The gradual weakening of the band at 1106 cm^−1^ and the gradual intensifications of the bands at 1550 and 1380 cm^−1^ observed in MOF-808 may be attributed to the generation of more Zr-OH species in the voids as the number of defects increases [36]. After the introduction of sulfate ions, additional vibration modes appear, particularly asymmetric and symmetric stretching vibrations. The new peaks detected at 1112 and 999 cm^−1^ in the MOF-808-SO_4_ spectrum correspond to O-S-O vibrations. Notably, as shown in Figure 2b, the bands at 1124 and 1047 cm^−1^ in the MOF-808-SO_4_ spectrum disappear because Zr-O-S bonds are formed after sulfonic acid group modification, confirming that sulfonic acid group was completely confined in the defective sites of MOF-808 [37,38].

**Figure 3:**
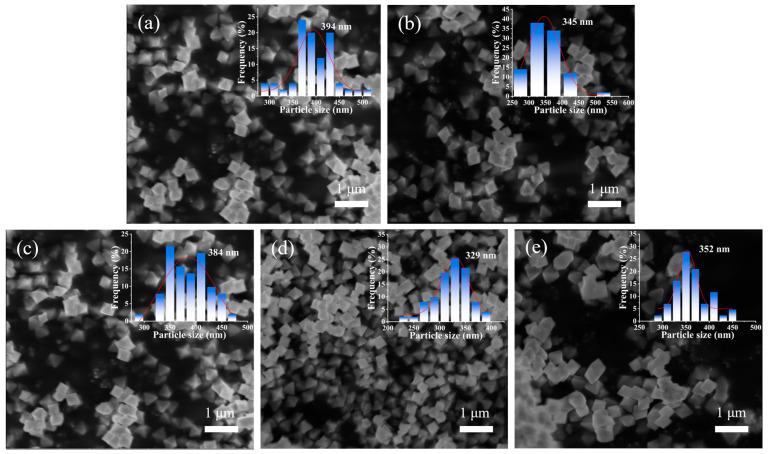
Scanning electron microscopy (SEM) images of MOF-808 (**a**), MOF-808-10% (**b**), MOF-808-20% (**c**), MOF-808-30% (**d**), MOF-808-40% (**e**).

**Figure 4:**
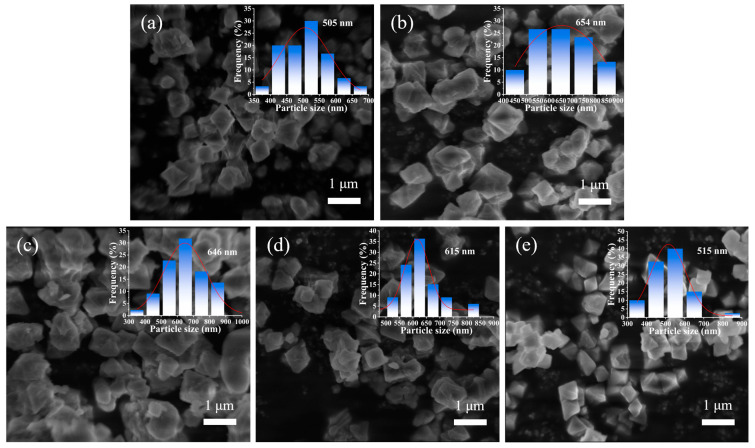
Scanning electron microscopy (SEM) images of MOF-808-SO_4_ (**a**), MOF-808-10-SO_4_ (**b**), MOF-808-20-SO_4_ (**c**), MOF-808-30-SO_4_ (**d**), MOF-808-40-SO_4_ (**e**).

SEM analysis was conducted to evaluate the morphological evolution and particle size distribution of the samples before and after sulfation. As shown in Figure 3 and Figure 4, all MOF-808-X samples exhibit regular octahedral morphology. Statistical analysis based on at least 100 particles per sample (with distribution histograms added directly onto each SEM image, see revised Figure 3 and Figure 4) shows that MOF-808-X (defect ratio 0-40%) has particle sizes mainly in the range of 300–400 nm. After sulfation, MOF-808-40-SO_4_ exhibits a larger particle size of approximately 515 nm. This increase is not due to simple aggregation but can be reasonably explained by the Ostwald ripening mechanism; in the acidic H_2_SO_4_ medium, smaller particles with higher surface energy dissolve preferentially, and the dissolved species redeposit onto larger, more stable particles, leading to an overall increase in average particle size. The unique octahedral morphology is largely maintained, likely due to the corrosive effect of H_2_SO_4_ on the crystal surface [39,40,41].

**Figure 5:**
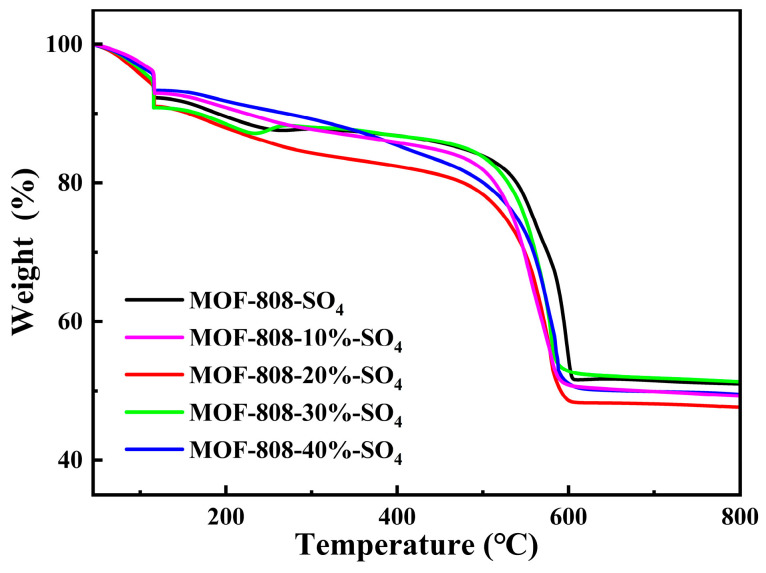
TG patterns of all MOF-808-SO_4_ samples.

The thermal stability of the prepared catalysts was characterized by thermogravimetric analysis, and the results are shown in Figure 5. All samples were vacuum-dried at 110 °C for 10 h prior to testing to remove physically adsorbed solvent molecules within the pores. The thermogravimetric curves reveal that the sulfated MOF-808 series catalysts exhibit a typical three-stage weight-loss behavior. The first weight loss of approximately 8% below 120 °C is assigned to the removal of surface and pore-physiosorbed solvents, including water, ethanol, and DMF [42]. The second slow weight loss in the range of 120–300 °C corresponds to further desorption of residual encapsulated solvents. The third weight loss starting near 520 °C originates from the thermal collapse and decomposition of organic ligands in the MOF framework. Notably, the mass remains nearly constant over the wide range of 300–520 °C, demonstrating excellent thermal stability of the catalysts. Overall, the sulfated MOF-808 materials maintain structural integrity below 520 °C, far exceeding the typical operating temperature of esterification, and thus satisfy the requirements for practical catalytic application.

The ligand composition and structural defects of the prepared samples were further quantified by ^1^H NMR (Figures S1–S5) and TGA calculation (Table 1 and Figure S6). With the increase in BDC feeding ratio, the BTC/BDC molar ratio gradually decreases, and the ligand defect concentration increases and reaches the highest value of 56.20% at 40% BDC. These results indicate that the introduction of BDC leads to an increase in ligand deficiency. The data are consistent with partial BDC incorporation, while excess BDC may also act as a modulator during defect formation. This inference is well consistent with the phenomenon that the actual BDC/(BTC + BDC) molar ratios are lower than the initial feeding ratios. The above effects determine the final defect concentration listed in Table 1. No significant difference is observed in the thermogravimetric curves of samples with different ligand ratios and defect levels, indicating that ligand deficiency does not significantly affect the thermal stability of the material.

**Figure 6:**
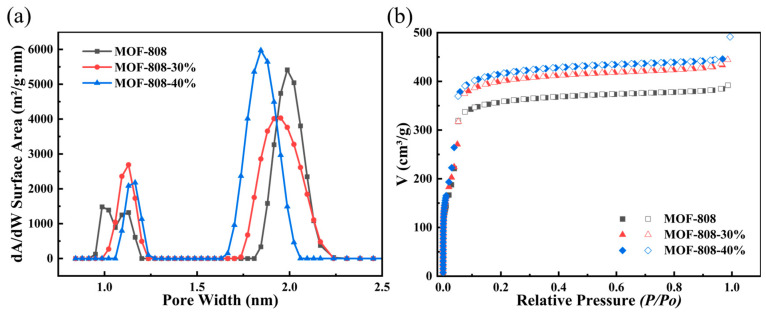
Pore size distribution curves (**a**); N_2_ sorption isotherms of MOF-808, MOF-808-30% and MOF-808-40% (**b**).

The porous characteristics of MOF-808, MOF-808-30% and MOF-808-40% were investigated by N_2_ adsorption–desorption measurements. Figure 6 shows the N_2_ adsorption–desorption isotherms of the defective sites activated MOF-808, MOF-808-30% and MOF-808-40%at 77 K. All samples exhibit typical type I adsorption isotherms [43], which are characteristic of microporous materials. As shown in Figure 6b, with increasing H_2_BDC substitution ratio, the N_2_ uptake at low relative pressure (P/P_0_ < 0.1) increases significantly, indicating a rise in the number of micropores. The corresponding pore size distribution curves (Figure 6a) reveal that the pore diameters of all samples are mainly in the range of 1.0–2.0 nm, falling within the microporous regime. Notably, as the defect degree increases, the pore size distribution becomes more homogeneous and more uniform, and the differential pore volume gradually increases. This suggests that defect engineering introduces additional micropores and leads to a more concentrated size distribution. Specifically, the BET surface areas and total pore volumes of MOF-808-30% and MOF-808-40% are all higher than those of the defect-free parent MOF-808 (Table 2), and with increasing BDC ratio, the BET surface area increases monotonically from 1679.3 m^2^/g to 2377.3 m^2^/g. These results collectively confirm that the generation of missing linker defects not only increases the total number of micropores but also renders the pore size distribution more homogeneous [29,44,45].

### 2.2. Surface Properties of the MOF-808

**Figure 7:**
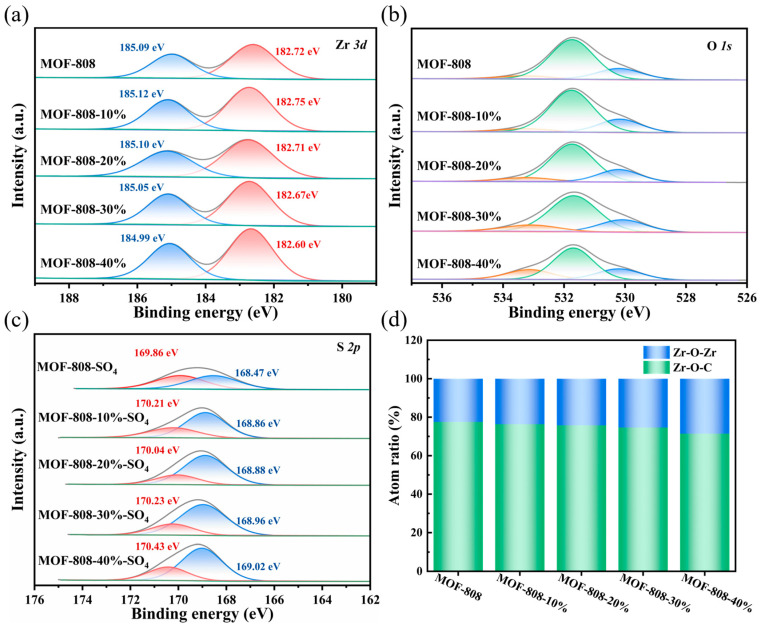
Zr 3d (**a**) and O 1s (**b**) XPS spectra of all MOF-808 samples; S 2p (**c**) XPS spectra of all MOF-808-SO_4_ samples; the contents of two types of oxygen groups calculated from the peak area of two types of oxygen (**d**).

The surface properties, such as acid density and surface species, of a catalyst are key factors determining its catalytic activity, selectivity, and stability. XPS was employed to investigate the chemical states and coordination environments of the elements within the framework. Figure 7 shows the high-resolution XPS spectra of Zr 3d and O 1s for five MOF-808 samples (MOF-808, MOF-808-10%, MOF-808-20%, MOF-808-30%, and MOF-808-40%), with the substitution ratio of isophthalic acid increasing successively. In the Zr 3d spectra, the binding energy of Zr 3d_5_/_2_ progressively decreases as the substitution ratio increases, dropping from 182.75 eV for MOF-808-10% to 182.71 eV for MOF-808-20%, 182.67 eV for MOF-808-30%, and further to 182.60 eV for MOF-808-40%. This monotonic shift toward lower binding energy suggests that ligand deficiency enhances the electron density around the Zr centers [46,47,48]. Furthermore, the high-resolution O 1s XPS spectra exhibit distinct variations among the MOF-808 samples. Three peaks are resolved at approximately 533.0, 531.8, and 530.0 eV, attributed to adsorbed oxygen (hydroxyl groups and adsorbed water), oxygen from coordinated carboxylate groups (Zr-O-C), and bridging oxygen (Zr-O-Zr), respectively [49]. As the BDC doping ratio increases, the peak intensity at around 530 eV shows a progressive enhancement (Figure 7a). Assuming the structural integrity of the Zr-O clusters (i.e., a constant number of Zr-O-Zr species), the relative content of Zr-O-C groups progressively decreases (Figure 7d), while the proportion of missing linkers increases with higher BDC addition. The high-resolution S 2p spectra (Figure 7c) verify the successful introduction of sulfur species after sulfation treatment. All sulfated samples show two characteristic peaks of S 2p_1_/_2_ and S 2p_3_/_2_ in the range of 168–171 eV, corresponding to the +6-oxidation state of sulfate groups. Notably, the binding energy of S 2p shifts progressively to higher values with increasing BDC ratio, indicating that sulfate groups are strongly chemically coordinated to the coordinatively unsaturated Zr sites in the defective MOF framework, rather than being physically adsorbed. This strong coordination interaction ensures the stable immobilization of sulfate acid sites, which is beneficial to the enhancement of catalytic activity and stability.

**Figure 8:**
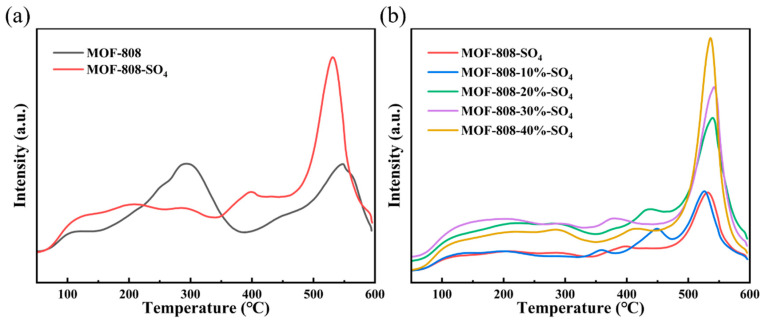
NH_3_-TPD profiles of the prepared catalysts: (**a**) comparison between MOF-808 and MOF-808-SO_4_; (**b**) acidity comparison of MOF-808-SO_4_ catalysts with different ligand ratios.

The surface acidity of the catalyst is a crucial factor determining its performance in the esterification reaction. To investigate the effects of sulfation modification and defect engineering on the acid properties of MOF-808, a series of catalysts were characterized by NH_3_-TPD, and the results are shown in Figure 8. Figure 8a compares the NH_3_-TPD curves of MOF-808 and MOF-808-SO_4_. Both samples exhibit ammonia desorption peaks attributed to weak acid sites in the low-temperature region (50–200 °C), and the desorption curves largely overlap, indicating that the sulfation treatment has little effect on the weak acid sites of the catalyst. However, in the medium- and high-temperature region (200–500 °C), the desorption signal of MOF-808-SO_4_ is significantly stronger than that of MOF-808, especially in the range of 400–500 °C. This difference is attributed to the successful introduction of sulfate groups, which form strong Brønsted acid sites (-HSO_4_) on the zirconium clusters, significantly increasing the density of moderately strong and strong acid sites. When the temperature rises above 500 °C, the desorption curves of the two samples gradually converge, indicating that the sulfation modification mainly affects the moderately strong and strong acid sites and contributes limitedly to the acid centers at extremely high temperatures.

Figure 8b shows the NH_3_-TPD curves of sulfated MOF-808-SO_4_ catalysts with different defect ratios (0%, 10%, 20%, 30%, 40%). As the ratio of defect ligands (BDC) increases, the intensity of ammonia desorption signals in all temperature ranges shows a gradually increasing trend, indicating that defect engineering effectively enhances the density of acidic sites on the catalyst.

In the low-temperature region, the weak acid site peaks increase slightly, which is attributed to the rising number of coordinatively unsaturated Zr clusters that act as Lewis acid centers and provide additional ammonia adsorption sites. More notably, in the medium- and high-temperature regions, the desorption signals of moderately strong and strong acid sites increase significantly with higher defect ratio. This enhancement is rationalized by the increased availability of anchoring sites for sulfate groups, enabling the formation of stronger Brønsted acid sites (-HSO_4_) [50,51].

To quantitatively confirm this trend, the total acidities of all MOF-808-SO_4_ samples were determined by titration. As listed in Table S1, the acidity order (MOF-808-SO_4_ < MOF-808-10-SO_4_ < MOF-808-20-SO_4_ < MOF-808-30-SO_4_ < MOF-808-40-SO_4_) matches perfectly with the NH_3_-TPD results. The H^+^ concentration of defective MOF-808-SO_4_ ranges from 2.90 to 4.31 mmol/g, with MOF-808-40-SO_4_ possessing the highest acidity. The introduction of BDC provides more unsaturated Zr sites for -HSO_4_ grafting, and the consistent increase in both NH_3_-TPD signal and titration-derived H^+^ concentration with defect density strongly supports that defect engineering promotes the formation of abundant strong Brønsted acid sites.

### 2.3. Catalytic Performance of MOF-808-SO_4_

**Figure 9:**
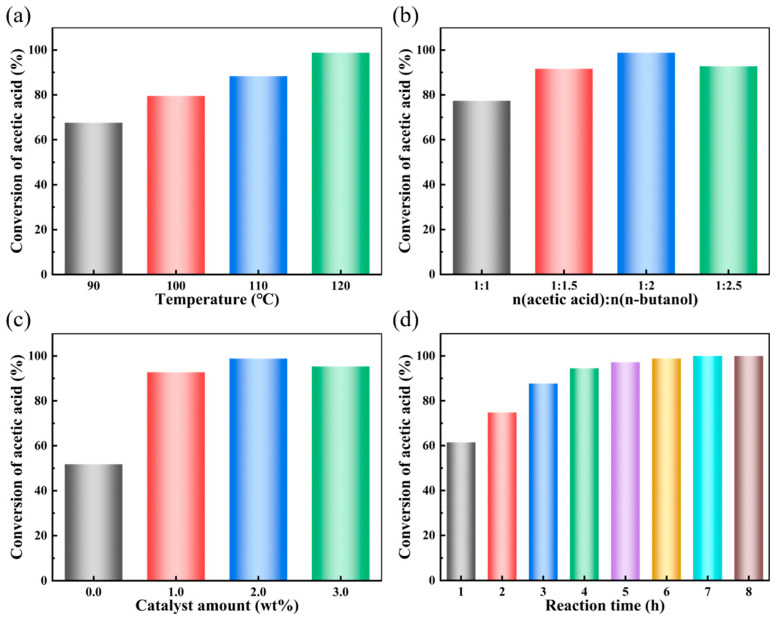
Impacts of various reaction conditions on the conversion of acetic acid over MOF-808-40-SO_4_: (**a**) reaction temperature, (**b**) acetic acid/n-amyl alcohol molar ratio, (**c**) catalyst amount, and (**d**) reaction time.

The performance of the esterification reaction is significantly affected by the reaction conditions. To determine the optimal reaction conditions for the catalytic esterification of acetic acid with n-butanol, MOF-808-40-SO_4_ was selected as the catalyst. This study systematically investigated the effects of reaction temperature, acid-to-alcohol molar ratio, catalyst dosage, and reaction time on the conversion of acetic acid. All optimization experiments were carried out sequentially using the single-factor variable method, and the results are shown in Figure 9.

The influence of reaction temperature on catalytic performance is shown in Figure 9a. In this set of experiments, the catalyst dosage was fixed at 2.0 wt%, the acid-to-alcohol molar ratio was 1:2, and the reaction time was 6 h. The change in acetic acid conversion was investigated in the temperature ranges from 90 to 120 °C. As the reaction temperature increases, the conversion of acetic acid shows a continuous upward trend, exhibiting the characteristics of a typical endothermic reaction. When the temperature increases from 90 to 120 °C, the conversion significantly increases from 56% to nearly 100%. The increase in the reaction temperature increases the collision frequency of reactant molecules and mass transfer rate, thereby accelerating the esterification reaction. Considering catalytic efficiency and energy consumption comprehensively, 120 °C is determined as the optimal reaction temperature and used in subsequent optimization experiments.

Based on the optimal reaction temperature (120 °C), with the catalyst dosage fixed at 2.0 wt% and reaction time at 6 h, the influence of acid-to-alcohol molar ratio on the esterification reaction was further investigated. The results are shown in Figure 9b. When the acid-to-alcohol molar ratio increases from 1:1 to 1:1.5 and then to 1:2, the conversions of acetic acid continuously and significantly improve, reaching 77.4%, 91.7%, and 98.8%, respectively. An appropriate excess of n-butanol not only facilitates the dispersion of the catalyst but also promotes the forward shift in the esterification reaction equilibrium. However, when the acid-to-alcohol molar ratio is further increased to 1:2.5, the conversion decreases to 92.8%. This may be due to the excess alcohol diluting the concentration of acid centers on the catalyst surface, reducing the probability of effective contact between the substrate and the active sites. Therefore, the optimal acid-to-alcohol molar ratio is identified to be 1:2, which is subsequently employed in optimization experiments.

Based on the optimal conditions (temperature: 120 °C, acid-to-alcohol ratio: 1:2), the reaction time was fixed at 6 h to investigate the influence of catalyst dosage on the esterification reaction and the results are displayed in Figure 9c. When the catalyst dosage is raised from 0.0 wt% to 2.0 wt%, acetic acid conversion significantly increases to 98.8% with gradually increased dosage. The increase in catalyst dosage provides more active sites available for the reaction—especially the strong Brønsted acid centers formed by the anchoring of sulfate groups at defect sites—which effectively promote the activation of substrate molecules. However, when the dosage is further increased to 3.0 wt%, the conversion is slightly decreased to 95.3%. The reason for this phenomenon is that an excessively high catalyst concentration may result in an increase in the viscosity of the reaction system, hindering effective mass transfer between the reactants and the catalyst. In conclusion, 2.0 wt% is demonstrated as the optimal catalyst dosage.

Under the above optimization conditions (temperature: 120 °C, acid-to-alcohol ratio: 1:2, catalyst dosage: 2.0 wt%), the influence of reaction time on the esterification reaction was investigated, and the results are shown in Figure 9d. Within the first 4 h of the reaction, the conversion increased rapidly, reaching nearly 95% at 4 h; it reached 98.83% at 6 h, and, thereafter, further extending the reaction time led to no significant change in conversion. Considering production efficiency, the optimal reaction time was determined to be 6 h for balancing catalytic activity and economic efficiency of the catalyst.

In summary, the optimal reaction conditions for the esterification of acetic acid and n-butanol catalyzed by MOF-808-40-SO_4_ are as follows: reaction temperature of 120 °C, catalyst dosage of 2.0 wt%, acid-to-alcohol molar ratio of 1:2, and reaction time of 6 h. Under these conditions, the conversion of acetic acid can approach 100%, fully demonstrating the excellent performance of the sulfated MOF catalyst constructed by defect engineering in the green esterification reaction.

**Figure 10:**
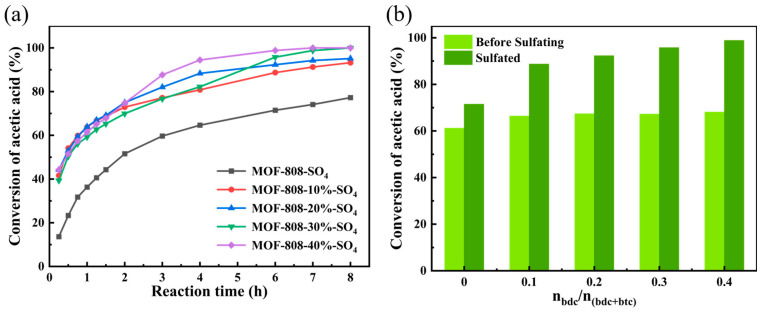
Effect of defect engineering (**a**) and sulfation modification (**b**) on the esterification performance of MOF-808 catalysts.

Under the optimized reaction conditions (temperature: 120 °C, catalyst dosage: 2.0 wt%, acid-to-alcohol molar ratio: 1:2), the variation in the esterification reaction performance of MOF-808-SO_4_ catalysts with different ligand ratios with reaction time was investigated, as shown in Figure 10a. As the reaction time extends, the acetic acid conversion of all catalysts gradually increases and tends to reach equilibrium after 6 h. The activity differences among catalysts with different ligand ratios are evident, and the order is 40% > 30% > 20% > 10% > 0%. Among them, MOF-808-40-SO_4_ exhibits the best catalytic performance at each time point, with a conversion of 98.8% after 6 h of reaction, while the non-defect-modified MOF-808-SO_4_ has only 71.5% acetic acid conversion.

The catalytic activity of the samples before and after sulfation treatment is compared in Figure 10b. Defect engineering via ligand substitution alone (without sulfation) had only a modest promotion effect, with acetic acid conversion increasing from 61% to 68% as the BDC ratio increased. In contrast, sulfation treatment led to a remarkably significant enhancement for all samples, with conversion increased by more than 20% compared to their unsulfured counterparts. These results clearly demonstrate that the dramatic improvement in catalytic activity after sulfation (from ~71% to 98.8% for the 40% defect sample) is primarily driven by the introduction of strongly coordinated sulfate groups at defect sites, rather than by the increase in BET surface area. XPS S 2p spectra (Figure 7c) confirm that with increasing defect density, the binding energy of sulfate shifts to higher values, indicating strong chemical coordination to coordinatively unsaturated Zr sites. Only after anchoring sulfate groups to defect sites did the activity increase drastically, confirming that the strong Brønsted acid sites formed via sulfate coordination are the real origin of catalysis, while porosity enhancement plays a supporting, not decisive, role.

The catalytic activity increases with increasing ligand ratio, which is attributed to the fact that the defect sites provide more anchoring sites for sulfate groups, promoting the formation of strong Brønsted acid centers (-HSO_4_). The XPS results further indicate that higher defect density leads to stronger sulfate coordination, which is consistent with both NH_3_-TPD signal and titration-derived H^+^ concentration. Thus, the density of acid centers shows a monotonically increasing trend with ligand ratio, in good agreement with the catalytic performance. Notably, the highest activity of the MOF-808-40-SO_4_ sample indicates that appropriate introduction of defects can maximize the density of effective acid centers while maintaining the integrity of the framework structure, thereby achieving the best esterification catalytic efficiency.

**Figure 11:**
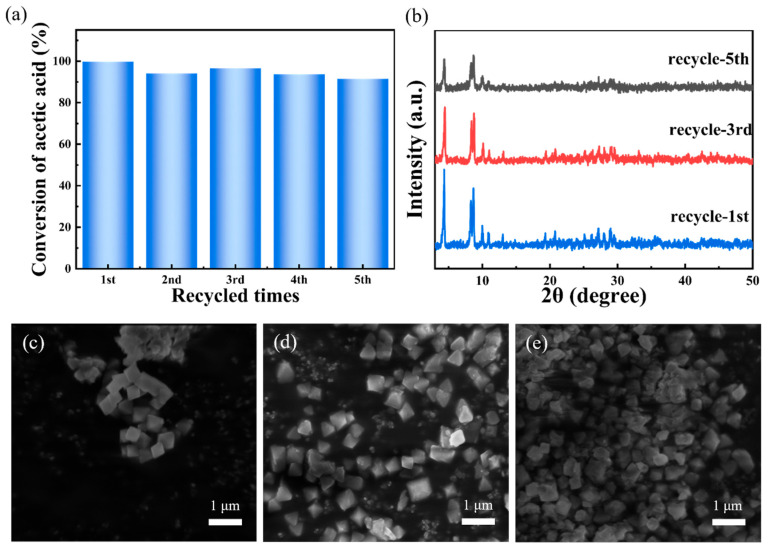
(**a**) Reusability of MOF-808-SO_4_-40%; (**b**) PXRD patterns; and (**c**–**e**) SEM images of MOF-808-SO_4_-40%: (**c**) recycle-1st, (**d**) recycle-3rd, and (**e**) recycle-5th.

To evaluate the catalytic stability of defective MOF-808-SO_4_, MOF-808-40-SO_4_ was selected as an example for cycling tests. After each experiment, the catalyst in the reaction solution was centrifuged. The collected catalyst was washed with ethanol, dried under vacuum conditions at room temperature, regenerated with sulfuric acid of the same concentration, and then used for the next experiment. A total of five cycling experiments were carried out, leading to an overall downward trend in acetic acid conversion (Figure 11a). For comparison, the cycling test results of the catalyst without sulfuric acid regeneration treatment are shown in Figure S7. In the fifth cycling experiment, the conversion decreases by approximately 7%. The possible reason for the decrease in catalytic activity is that polar n-butanol may damage the structure of the catalyst. The SEM image also shows that part of the framework structure has collapsed (Figure 11c–e), indicating that the stability of the catalyst is a key factor in the reaction.

### 2.4. MOF-808-40-SO_4_ for Extended Esterification Reactions and Comparison

Considering the remarkable activity exhibited by the MOF-808-40-SO_4_ catalyst, we investigated its esterification performance with acetic acid and various alcohols. MOF-808-40-SO_4_ demonstrated significant catalytic activity in the esterification reactions of acetic acid with n-pentanol, n-hexanol, n-heptanol, and n-octanol, achieving conversions above 93% under the optimized conditions listed in Table 3.

Table 4 compares the catalytic performance of MOF-808-40-SO_4_ with those of various reported MOF-based catalysts toward the esterification of acetic acid with different alcohols. Most reported MOF catalysts either suffer from low acid density, insufficient exposed active sites, or limited structural stability under esterification conditions, leading to relatively low catalytic efficiency. In contrast, the defective MOF-808-40-SO_4_ catalyst exhibits significantly enhanced catalytic activity, highlighting its promising potential as an efficient solid acid catalyst for the esterification of acetic acid with diverse alcohols.

## 3. Experimental Section

### 3.1. Materials

All the chemicals were of analytical grade and used without any further purification. Isophthalic acid (BDC, ≥99%), N,N-dimethylformamide (DMF, 99.5%), formic acid (CH_2_O_2_, ≥99%), ethanol (C_2_H_6_O, ≥99.7%), and acetic acid (C_2_H_4_O_2_, 99.5%) were obtained from Shanghai Aladdin Biochemical Technology Co., Ltd. (Shanghai, China). n-Butanol (C_4_H_10_O, ≥99.8%), zirconium oxychloride octahydrate (ZrOCl_2_·8H_2_O, ≥99%) and phenolphthalein were bought from Sinopharm Chemical Reagent Co., Ltd. (Shanghai, China). Sulfuric acid (H_2_SO_4_) was purchased from Xilong Scientific Co., Ltd. (Shanghai, China) and 1,3,5-benzenetricarboxylic acid (BTC, 99%) came from Shanghai Jinyi Nuo Biotechnology Co., Ltd. (Shanghai, China). Sodium chloride (NaCl, ≥99.5%) was bought from Shanghai Ling Feng Chemical Reagent Co., Ltd. (Shanghai, China). Sodium hydroxide (NaOH, ≥98%) was purchased from Shanghai Macklin Biochemical Co., Ltd. (Shanghai, China).

### 3.2. Catalysts Preparation

#### 3.2.1. Synthesis of MOF-808-X

MOF-808 was first prepared as super acid catalyst from a previous report [37] and defective MOF-808 was synthesized by the following approaches: We chose five molar concentrations (X) of BDC/(BTC + BDC) ranging from 0% to 40% (X = 0%, 10%, 20%, 30%, 40%) during preparation, and the resulting samples were denoted as MOF-808-X, where BDC was the organic defect ligand to regulate defect sites of MOF-808. Typically, MOF-808-10% was synthesized as follows: ZrOCl_2_·8H_2_O (3 mmol), BDC (0.1 mmol), and BTC (0.9 mmol) were dissolved in a mixture of DMF and formic acid (45 mL/45 mL). The homogeneous solution was transferred into a Teflon-lined stainless-steel autoclave, which was then sealed and heated at 130 °C for 24 h to facilitate crystal growth. Crystalline material (MOF-808-10%) was collected and washed with DMF and ethanol for three days, respectively.

#### 3.2.2. Synthesis of MOF-808-SO_4_-X

The synthesized MOF-808-X (0.5 g) was immersed in 50 mL of the sulfuric acid solution (0.5 mol/L) and magnetically stirred for 24 h. The solid was separated by centrifugation and washed three times with deionized water and anhydrous ethanol, respectively. Then the resulting product was dried in a vacuum oven at 120 °C for 12 h for future applications. The catalyst was denoted as MOF-808-X-SO_4_.

### 3.3. Characterizations

Powder X-ray diffraction (PXRD) patterns of all samples were obtained on a Smartlab X-ray diffractometer (Rigaku Corporation, Akishima, Tokyo) with Cu Kα radiation (λ = 1.5418 Å) at room temperature and the 2θ range of 3–50°. Thermogravimetric analysis (TGA) curves were performed on a PerkinElmer Pyris 1 TGA analyzer (PerkinElmer Inc., Waltham, MA, USA). Samples were heated from 30 °C to 800 °C at a heating rate of 15 °C min^−1^ under an air flow of 40 mL/min. N_2_ absorption/desorption analyses were carried out on a Micromeritics 3Flex (Micromeritics Instrument Corporation, Norcross, GA, USA) Highest-performance gas adsorption analyzer via Brunauer-Emmett-Teller (BET) and Barrett-Joyner-Halenda (BJH) methods. Prior to the N_2_ absorption/desorption measurements, all materials were degassed at 100 °C for 12 h under dynamic vacuum.

NH_3_ temperature-programmed desorption (NH_3_-TPD) was conducted on a Micromeritics AutoChem II 2920 (Micromeritics Instrument Corporation, Norcross, GA, USA) automatic chemisorption analyzer to investigate the surface acidity of the catalysts. In a typical experiment, approximately 100 mg of the catalyst was loaded into a U-shaped quartz reactor and pretreated under an argon flow at 120 °C for 1 h to remove adsorbed species. After the reactor was cooled to room temperature, the gas flow was switched to a 10 vol% NH_3_/Ar mixture for 1 h to allow probe molecule adsorption. Subsequently, the gas flow was switched back to high-purity Ar to purge physically adsorbed NH_3_ and residual gas from the reactor. The desorption signal was then recorded using a thermal conductivity detector (TCD) while the temperature was ramped from 30 °C to 650 °C at a heating rate of 10 °C/min. 1H NMR spectra of ligands in all MOF-808 samples digested in DMSO-d6 were recorded on a Bruker Advance spectrometer (400 MHz, Bruker Corporation, Billerica, MA, USA). X-ray photoelectron spectroscopy (XPS) spectra were collected on a Thermo Scientific K-Alpha electron spectrometer (Thermo Fisher Scientific Inc., Wilmington, DE, USA) using monochromatic Al Kα radiation (hν = 1486.6 eV) as the excitation source. All binding energies were calibrated by referencing the adventitious carbon C 1s peak at 284.8 eV to correct for charging effects.

The acid amount of the catalysts was determined by titration. Typically, a small amount of sample was added to a NaCl solution containing excessive Na, and allowed to undergo ion exchange for 24 h. Using phenolphthalein as the indicator, the solution was titrated with 0.1 mol/L NaOH until the color changed from colorless to pale pink, which was taken as the endpoint. The acid amount was then calculated based on the volume of NaOH consumed.

### 3.4. Esterification Reaction

The esterification reaction was conducted in a three-necked, round-bottom flask with a volume of 50 mL. A condenser was attached to the top of the flask to prevent the loss of components with anti-backflow suction. The solid acid catalyst and a fixed molar ratio of acetic acid and alcohol were loaded into the flask. Once the seal was confirmed, the flask was placed in a preheated oil bath at a specific temperature under continuous stirring. The acid-to-alcohol ratio was within the range of 1:1 to 1:2.5, and the temperature was within the range of 90 to 120 °C. The catalyst was employed in quantities of 1 to 3% of the total mass of acetic and alcohol. Subsequent to the initiation of the reaction, the sample was collected at regular intervals. A precise quantity of 0.5 mL of the liquid sample was taken using a syringe, which was immediately cooled in order to prevent further reaction, and filtered using a needle filter.

### 3.5. Product Analysis

The concentrations of acetic acid and other products were determined using an Agilent 7890A gas chromatograph (Agilent Technologies Inc., Santa Clara, CA, USA) equipped with a flame ionization detector (FID) and an HP-FFAP chromatographic column (30 m × 0.32 mm × 0.25 μm). The analysis was conducted under the following conditions: nitrogen gas with a purity of 0.999 was used as a carrier gas, and the injector and detector temperatures were maintained at 250 °C. The initial column temperature of 60 °C was increased to 90 °C at a rate of 30 °C/min, kept for 2 min, and then rapidly heated to 230 °C at a rate of 30 °C/min. Toluene was used as an internal standard for quantitative analysis. The conversion of acetic acid (*x*_A_) was calculated using the equation below:

$$x_{A}\;=\;\frac{Mole\;of\;acetic\;acid\;reacted}{Mole\;of\;acetic\;acid\;initially}$$

## 4. Conclusions

In this study, a series of MOF-808-SO_4_ solid acid catalysts with different proportions of organic ligand defects were successfully prepared and applied to the esterification synthesis of n-butyl acetate. The influence of defect degree on catalyst structure, acidic sites, and catalytic performance was systematically investigated. The experimental results show that the MOF-808-SO_4_ catalyst with a ligand defect proportion of 40% exhibits optimal esterification catalytic performance. This is attributed to the appropriate pore size structure, sufficient strong Brønsted acid sites, favorable pore diffusion, and structural stability of the material at this defect degree, which effectively balances the relationship between active site exposure, substrate diffusion, and mass transfer, achieving excellent catalytic efficiency under optimized reaction conditions. The optimal catalyst was further extended to esterification reaction systems with primary alcohols of different carbon chain lengths. The results reveal that the catalyst displays excellent substrate applicability to various long-chain and short-chain primary alcohols, with acetic acid conversion reaching over 90%, demonstrating its good catalytic universality. This catalyst can serve as an efficient and general solid acid catalyst for the green synthesis of various fatty acid esters. In addition, this study extends from single-substrate reactions to multi-chain-length primary alcohol systems and preliminarily reveals the structure–activity relationship between the pore structure of defective MOF catalysts and alcohol molecules of different chain lengths, providing experimental support for the targeted design and performance regulation of porous MOF-based solid acid catalysts.

Nevertheless, we acknowledge that our pristine MOF-808 had a lower BET surface area than reported values, partly due to synthesis scale-up and reactant ratios. This limitation does not affect the main conclusions, as the relative improvements from defect engineering remain valid. To further strengthen the findings, future work will focus on: (1) preparing a highly porous pristine MOF-808 to validate the synergy between defect engineering and sulfation; and (2) determining the spatial origin of active sites.

## Figures and Tables

**Table 1:** Calculated residual mass fraction, framework molecular weight, ligand number, and defect concentration of different MOF-808 samples.

Sample	W_res_ (%)	BTC/BDC (Molar Ratio)	M_frame_ (g/mol)	L_real_	ΔL	Defect Concentration (%)
MOF-808-10%	53.77	5.797	1374.97	3.12	2.88	47.98
MOF-808-20%	54.48	2.778	1357.05	3.11	2.89	48.13
MOF-808-30%	54.83	1.646	1348.39	3.15	2.85	47.54
MOF-808-40%	59.31	1.569	1246.54	2.63	3.37	56.20

W_res_ is the residual ZrO_2_ mass fraction at high temperature, M_frame_ represents the calculated framework molecular weight, L_real_ refers to the actual coordinated ligand number per Zr_6_ cluster, and ΔL is the number of missing ligands.

**Table 2:** BET surface areas, pore volumes and pore diameters of MOF-808, MOF-808-30% and MOF-808-40%.

Sample Names	BET Surface Area (m^2^/g)	Total Pore Volume (cm^3^/g)	Average Pore Diameter (nm)
MOF-808	1679.3	0.607	1.58
MOF-808-30%	2129.3	0.689	1.29
MOF-808-40%	2377.3	0.757	1.27

**Table 3:** The activity of MOF-808-40-SO_4_ for esterification of acetic acid with various alcohols.

Catalyst	Reactants	Conditions	Conversion of Acetic Acid (%)
MOF-808-40-SO_4_	n-butanol	120 °C, n (n-butanol): n (acetic acid) = 2:1, 2.5 wt% catalyst, 6 h	98.0
MOF-808-40-SO_4_	n-amyl alcohol	120 °C, n (n-amyl alcohol): n (acetic acid) = 2:1, 2 wt% catalyst, 6 h	95.1
MOF-808-40-SO_4_	n-hexyl alcohol	120 °C, n (n-hexyl alcohol): n (acetic acid) = 2:1, 2.5 wt% catalyst, 6 h	93.5
MOF-808-40-SO_4_	n-heptanol	120 °C, n (n-heptanol): n (acetic acid) = 2:1, 2 wt% catalyst, 6 h	96.4
MOF-808-40-SO_4_	n-octanol	120 °C, n (n-octanol): n (acetic acid) = 2:1, 2 wt% catalyst, 6 h	97.3

**Table 4:** The activity of MOFs catalysts for esterification of acetic acid with various alcohols.

Catalyst	Reactants	Conditions	Conversion of Acetic Acid (%)	Ref.
HSO_3_-UiO-66	Acetic acid and n-butanol	70 °C, n (n-butanol): n (acetic acid) = 1:1, 2.2 wt% catalyst, 10 h	58.0	[52]
HSO_3_-MIL-53	Acetic acid and n-butanol	70 °C, n (n-butanol): n (acetic acid) = 1:1, 2.2 wt% catalyst, 10 h	58.0	[52]
HPW@UiO-67	Acetic acid and n-butanol	120 °C, n (n-amyl alcohol): n (acetic acid) = 2:1, 2.0 wt % catalyst, 5 h	72.3	[53]
MOF-808-40-SO_4_	n-butanol	120 °C, n (n-butanol): n (acetic acid) = 2:1, 2.5 wt% catalyst, 6 h	98.0	This work
MOF-808-40-SO_4_	n-amyl alcohol	120 °C, n (n-amyl alcohol): n (acetic acid) = 2:1, 2 wt% catalyst, 6 h	95.1	This work
MOF-808-40-SO_4_	n-hexyl alcohol	120 °C, n (n-hexyl alcohol): n (acetic acid) = 2:1, 2.5 wt% catalyst, 6 h	93.5	This work
MOF-808-40-SO_4_	n-heptanol	120 °C, n (n-heptanol): n (acetic acid) = 2:1, 2 wt% catalyst, 6 h	96.4	This work
MOF-808-40-SO_4_	n-octanol	120 °C, n (n-octanol): n (acetic acid) = 2:1, 2 wt% catalyst, 6 h	97.3	This work

## Data Availability

The data presented in this study are available in this article.

## Supplementary Materials

The following supporting information can be downloaded at: https://www.mdpi.com/article/10.3390/molecules31111908/s1, Figure S1: ^1^H NMR spectra of digested MOF-808; Figure S2: ^1^H NMR spectra of digested MOF-808-10%; Figure S3: ^1^H NMR spectra of digested MOF-808-20%; Figure S4: ^1^H NMR spectra of digested MOF-808-30%; Figure S5: ^1^H NMR spectra of digested MOF-808-40%; Figure S6: TG patterns of all MOF-808 samples; Figure S7: Reusability of all MOF-808-SO_4_ samples without sulfuric acid regeneration treatment; Table S1: The acidity of all MOF-808-SO_4_ samples.
