# Supplementary material for: Defect-Engineered MOF-808-SO4 as Efficient Solid Acid Catalysts for Esterification of n-Butyl Acetate
Source: Molecules. 2026 Jun 2;31(11):1908. doi: 10.3390/molecules31111908 (PMC13257938; doi:10.3390/molecules31111908)
Supplement: Supplementary file 1 [file molecules-31-01908-s001.zip › molecules-4258602-supplementary.pdf]

Supporting Information

Defect-Engineered MOF-808-SO_4 as Efficient Solid Acid Catalysts for Esterification of n-Butyl Acetate

Wei Cao, Lifang Chen *, Tingting Wang, Ke Wang, Zhen Song and Zhiwen Qi *

State Key Laboratory of Chemical Engineering and Low-Carbon Technology, School of Chemical Engineering, East China University of Science and Technology, 130 Meilong Road, Shanghai 200237, China

** Correspondence: lchen@ecust.edu.cn (L. Chen); zwqi@ecust.edu.cn (Z. Qi)*

Supplementary Figures

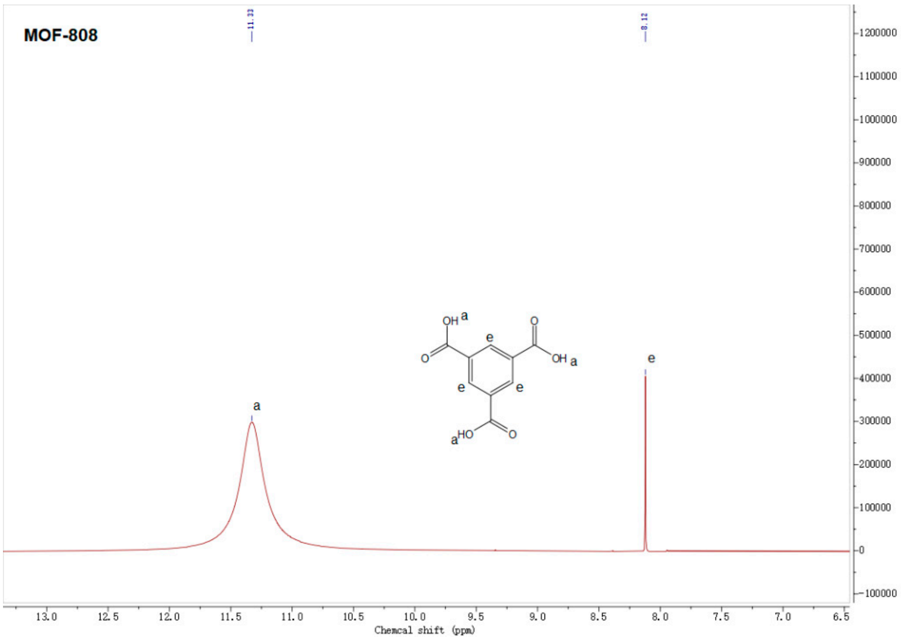

Figure S1. ^{1}H NMR spectra of digested MOF-808.

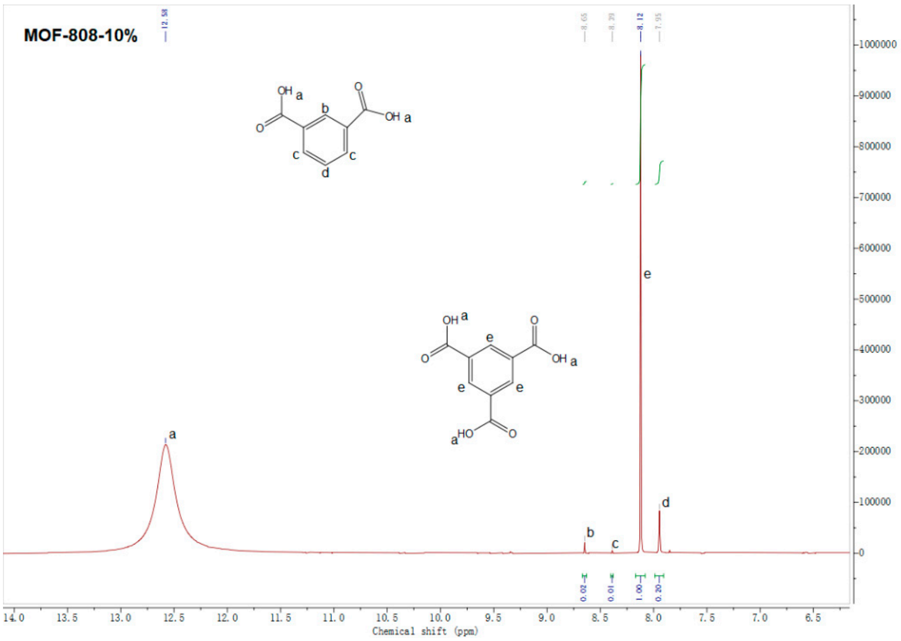

Figure S2. ^{1}H NMR spectra of digested MOF-808-10%.

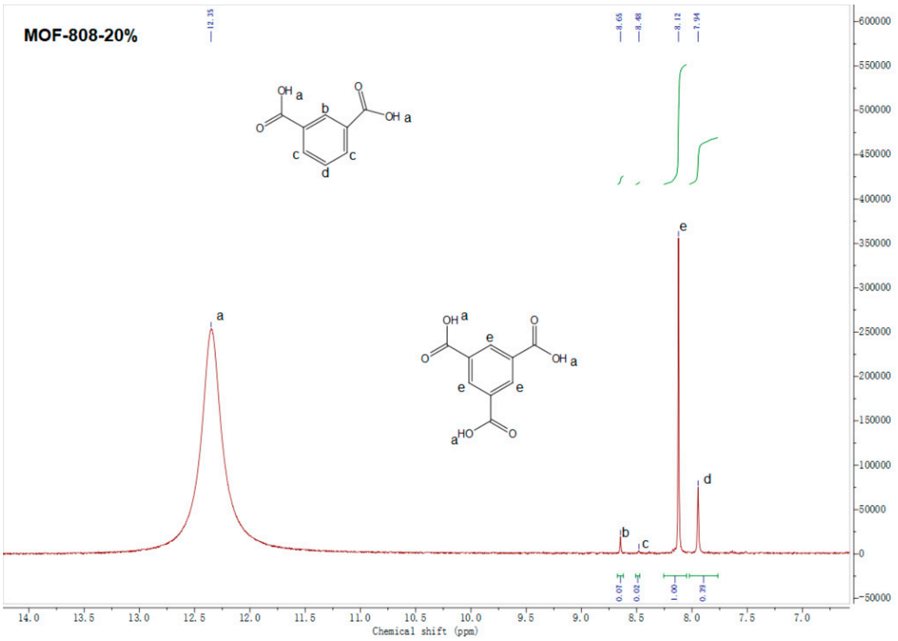

Figure S3. ^{1}H NMR spectra of digested MOF-808-20%

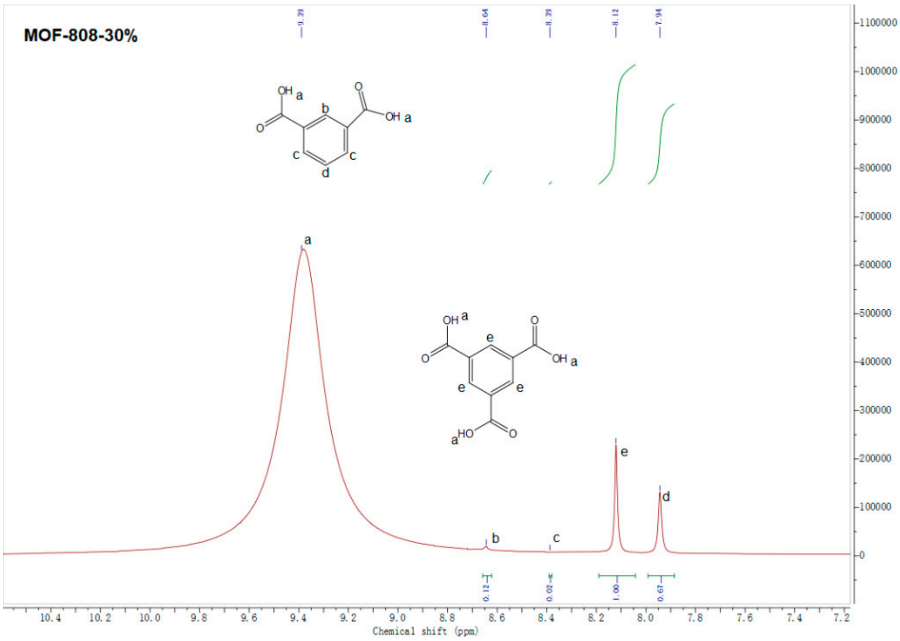

Figure S4. ^{1}H NMR spectra of digested MOF-808-30%

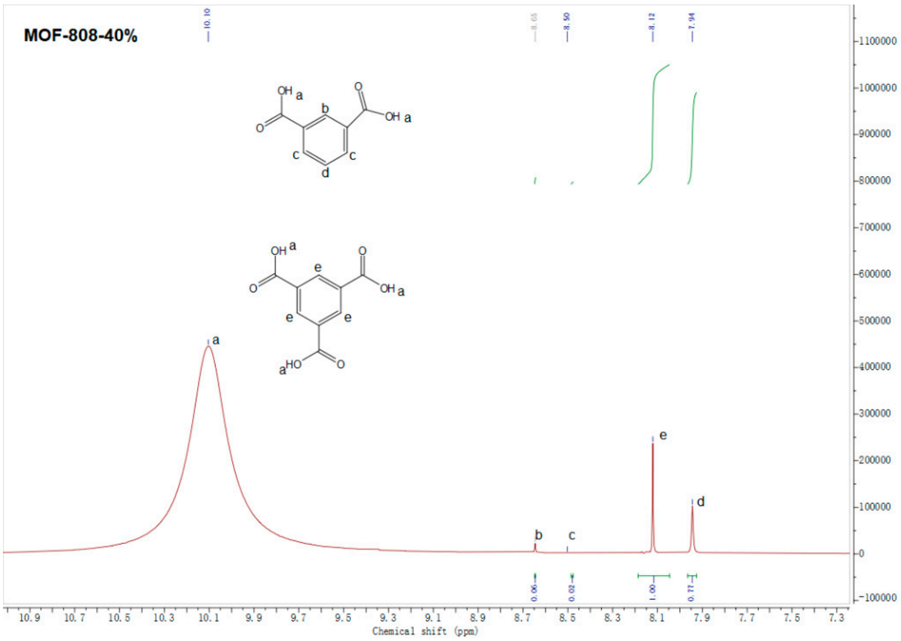

Figure S5. ^{1}H NMR spectra of digested MOF-808-40%

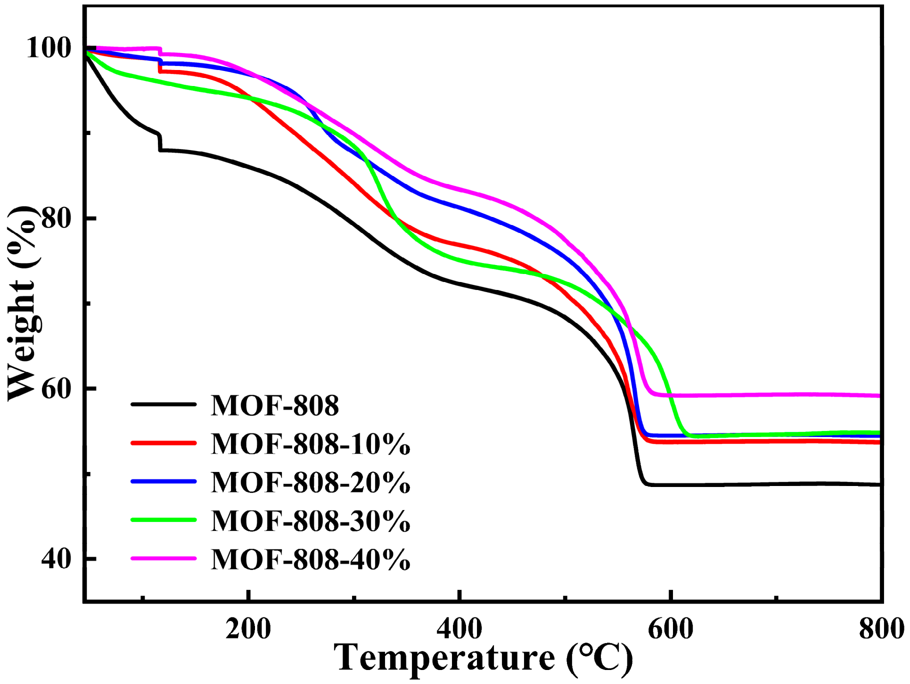

Figure S6. TG patterns of all MOF-808 samples.

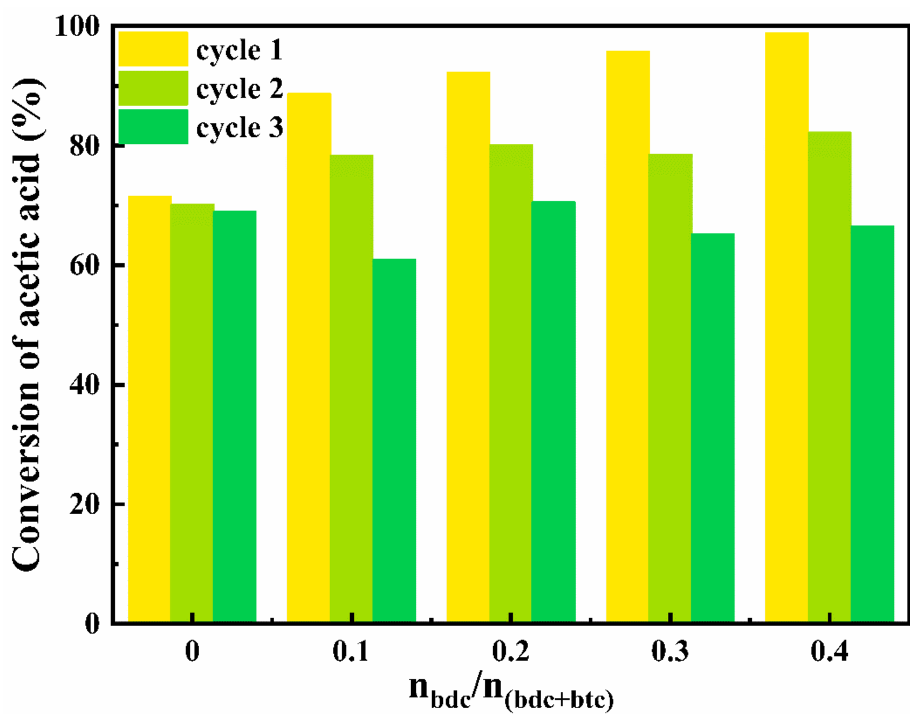

Figure S7. Reusability of all MOF-808-SO_4 samples without sulfuric acid regeneration treatment.

Table S1. The acidity of all MOF-808-SO_4 samples.

Catalyst	Acid density H^+ (mmol/g)
MOF-808-SO_4	2.90
MOF-808-SO_4-10%	3.58
MOF-808-SO_4-20%	3.84
MOF-808-SO_4-30%	3.95
MOF-808-SO_4-40%	4.31
